# Tumor Budding Should Be in Oral Cavity Cancer Reporting: A Retrospective Cohort Study Based on Tumor Microenvironment

**DOI:** 10.3390/cancers15153905

**Published:** 2023-08-01

**Authors:** Ayca Tan, Toros Taskin

**Affiliations:** 1 Department of Pathology, Manisa Celal Bayar University, Manisa 45030, Turkey; 2 Department of Pathology, Agri Training and Research Hospital, Agri 04200, Turkey; torostaskin@hotmail.com

**Keywords:** oral, squamous, carcinoma, invasion, budding, stroma

## Abstract

**Simple Summary:**

In our study, which started with the hypothesis that there is a histopathological marker that can be used to predict prognosis in oral squamous cell carcinomas, we found that tumor budding is quite significant. The fact that this finding will provide us with very important data in routine practice and play a key role in the treatment management of patients will be a significant finding and contribution to the literature.

**Abstract:**

The utility of histological grading, which is useful in predicting prognosis in many tumors, is controversial for oral squamous cell carcinoma (OSCC). Therefore, new histopathological parameters should be added to histopathology reports of OSCCs. The study aimed to evaluate the parameters of worst invasion pattern (WPOI) and tumor budding in patients with OSCC, to compare them with other histopathological parameters, clinical data and overall survival, and to evaluate these results within the literature. A total of 73 OSCC cases with excisional biopsies were included in this study. WPOI, tumor budding, cell nest size, tumor-stroma ratio, stromal lymphocyte infiltration and stroma type, as well as classical histopathological parameters, were evaluated on hematoxylin-eosin-stained sections. Perineural invasion, lymph node metastases, advanced stage, presence of more than five buds and single cell invasion pattern in univariate survival analyses are characterized by a shortened overall survival time. While there was no significant difference between WPOI results and survival in the survival analysis, WPOI 5 was associated with more frequent lymph node metastasis and advanced stage at the time of diagnosis compared to WPOI 4. We concluded that tumor budding and single-cell invasion should be considered prognostic histopathologic parameters in OSCC.

## 1. Introduction

Oral cancer is the 16th most common cancer in the world, and approximately 377,000 cases are diagnosed annually, and 70% of the cases are male [1]. Oral squamous cell carcinoma (OSCC) accounts for more than 90% of malignancies in the oral cavity [2]. Smoking, alcohol and smokeless tobacco are common risk factors [2,3]. HPV, an important etiological factor in oropharyngeal cancer is only seen in 3% of all cases [4].

The World Health Organization (WHO) classification and other major pathology guidelines recommend a three-tiered histological grading system for head and neck SCCs. The utility of histological grading, which is useful in predicting prognosis in many tumors, is controversial for OSCC. In this system, tumors are classified as well, moderately and poorly differentiated according to their features, such as the degree of keratinization, cytonuclear atypia and infiltration pattern [1]. The inadequate/insufficient relationship between histological grading and prognosis has led researchers to explore new and different histopathological parameters. In particular, parameters associated with the tumor microenvironment are thought to have a significant impact on tumor behavior and significantly affect OSCC prognosis [5].

The worst pattern of invasion (WPOI), which was first published by Jakobsson et al. in 1973, gained momentum in 2005 when Brandwein-Gensler et al. studied this entity. Many studies revealed that category 5 of the WPOI was a poor prognostic factor for either recurrence or survival, while histological grade could not predict the prognosis [6].

Tumor budding is defined as tumor clusters of less than five cells [7]. The prognostic importance of tumor budding has been recognized in colon cancers, and it is recommended to be reported in the College of American Pathologists (CAP) colon cancer guidelines; however, it is not yet reported in the oral cavity cancer guidelines. Studies in the literature showed that tumor budding is related to recurrence, lymph node metastasis and poor prognosis [8,9,10].

Tumor cell nest size is another parameter that predicts the prognostic parameters. It has been reported in many studies and different tumors that those with single-cell invasion have a worse prognosis [11,12,13,14].

Stromal changes, such as tumor-stroma ratio (stroma poor/stroma rich), stromal lymphocytic infiltration and stroma type (mature/immature), are also variables that can be useful to predict the lymph node metastasis, recurrence, distant metastases and prognosis [11,15,16,17,18,19].

This study aimed to compare WPOI, tumor budding, tumor cell nest size and stromal parameters with clinical data and overall survival in patients with OSCC and to evaluate these results within the literature. For this purpose, we aimed to evaluate these parameters and routine histopathological parameters in patients with OSCC who are registered in our archive and whose follow-up is regular and to determine whether there is a significant histopathological parameter in predicting the prognosis.

## 2. Materials and Methods

All of the cases located on the tongue and the other areas in the oral cavity diagnosed with squamous cell carcinoma were listed. Cases without follow-up information and having only incisional biopsies and also biopsies which were not suitable to calculate for depth of invasion and have no slides or blocks in the archive were excluded. A total of 73 cases with complete clinical information and histopathological data were included in the study.

Age, gender, tumor localization and tumor size were noted in pathology reports. The cases were re-evaluated for routine histopathological parameters according to WHOs 5th edition. The histological grade (differentiation), depth of invasion, surgical margin status, perineural invasion, lymphovascular invasion, lymph node metastasis, extranodal extension, size of extranodal extension and stage of the cases were noted. The differentiation at the deep invasive border of the tumor was evaluated for WHO histological grading [20]. Surgical margins closer than 1 mm were considered positive. TNM stage was reevaluated according to the current American Joint Committee on Cancer (AJCC) staging guidelines [21]. Locoregional recurrence, distant metastasis and follow-up status of the patients were obtained from the hospital database.

Parameters that are not mandatory in routine pathology reports, such as WPOI, tumor budding, tumor cell nest size, tumor-stroma ratio, stromal lymphocyte infiltration and stroma type, were classified and their contribution to prognosis was evaluated.

WPOI: The 5-tiered classification created by Brandwein-Gensler et al. was used in the evaluation of WPOI [6]. WPOI 1; broad pushing growth, WPOI 2; finger-like pushing growth or discrete large tumor islets with a stellate appearance, WPOI 3; invasive tumor islands containing more than 15 cells, WPOI 4; invasive tumor islands containing less than 15 cells, WPOI 5; tumor satellites at least 1 mm from the tumor at the tumor/host junction.

Tumor budding: Tumor budding is the presence of a single cancer cell or less than 5 cells in the tumor stroma. According to the guidelines published by the International Tumor Budding Consensus Conference (ITBCC), tumor budding should be evaluated on the invasive front using the ×200 objective. ITBCC uses 3 grades of grading for tumor budding, low (0–4 buds), medium (5–9 buds) and high (≥10 buds) [7]. In our study, the budless group was also evaluated and 4-stage grading was used.

Tumor cell nest size: The smallest invasive cell nest in the stroma was considered when evaluating the cell nest size. Clusters of more than 15 tumor cells were classified as large cell nests, 5–15 tumor cells as intermediate cell nests, 2–4 tumor cells as small cell nests and 1 tumor cell as single cell invasion [13].

Tumor-stroma ratio: All slides were scanned at low magnification (×40) to select an area with the highest amount of stroma on the invasive front and also cancer cells at all four sides of the field to calculate the tumor stroma ratio. After that, the selected area was scored as stroma-poor (<50%) or stroma-rich (≥50%) under higher magnification (×100) [22].

Stromal lymphocyte infiltration: Stromal lymphocyte infiltration was graded as low (0–30%), intermediate (31–60%), and high (>60%) based on the guidelines published by the International Immuno-Oncology Biomarker Study Group for the evaluation of tumor-infiltrating lymphocytes in solid tumors [23]. Cystic changes and necrotic areas were excluded.

Stroma type: Stroma type was classified into two categories as mature and immature. Mature stroma was composed of fine mature collagen and does not contain myxoid stroma or keloid-like collagen. The immature stroma was composed of fibrotic stroma with myxoid changes in at least one area at high magnification (×400) [16].

All data were analyzed with SPSS (Statistical Package for the Social Sciences) version 25 (IBM SPSS-30 day Free trial). The data were analyzed by Chi-square and Fisher’s exact test and then tested by logistic regression analysis. Kaplan–Meier method and a log-rank test were used for survival analysis. In the survival analysis, the hazard ratio (Hazard Ratio) and 95% Confidence Interval (CI) were calculated. The data that were significant in univariate analyzes were reanalyzed by creating a multivariate Cox regression risk model. The statistical significance was accepted as *p* < 0.05.

## 3. Results

The patient’s age ranged from 28 to 85 years (mean: 61.62, median: 65). The male/female ratio (1.02) was almost equal. Tumor size ranged from 0.3 cm to 10 cm (mean: 2.4 cm, median: 2 cm) and the depth of invasion was 0.2 mm to 40 mm (mean: 8.2 mm, median: 6 mm). The demographic and histopathological parameters were shown in Table 1.

**Figure 1:**
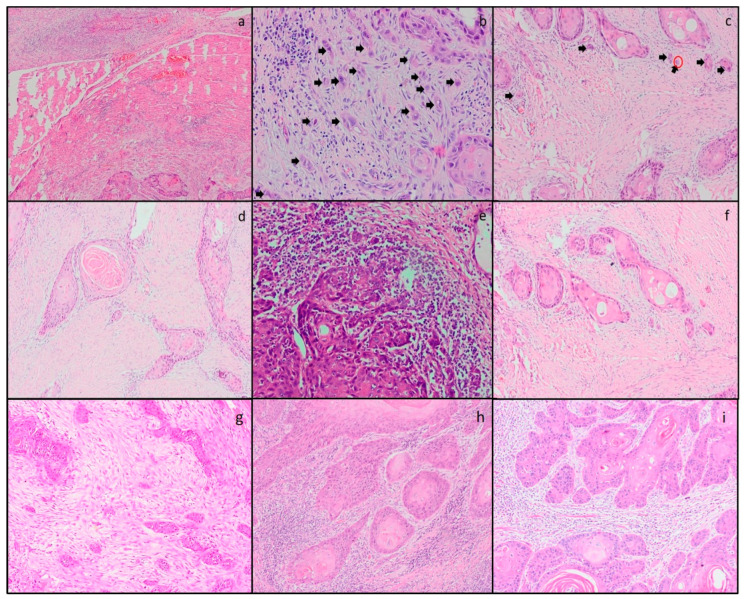
(**a**) WPOI 5; tumor satellites at least 1 mm from the tumor at the tumor/host junction (HEx40), (**b**) high tumor budding (≥10 buds) (tumor budding showed with arrows) and WPOI 4 (HEX400), (**c**) medium tumor budding (5–9 buds) (tumor budding showed with arrows) and single-cell invasion (showed with a red circle) (HEX200), (**d**) stroma-rich pattern and low (<30%) stromal lymphocyte infiltration and intermediate cell nests (cluster of 5–15 tumor cells) (HEX200), (**e**) high (>60%) stromal lymphocyte infiltration (HEX200), (**f**) mature stroma and single cell invasion and low tumor budding (1–4 buds) (HEX200), (**g**) immature stroma and small cell nests (cluster of 2–4 cells) (HEX200), (**h**) WPOI 3 and large cell nests (cluster of >15 cells) (HEX200), (**i**) stroma poor pattern and no budding (HEX200).

The distribution of histopathological parameters examined for prognostic significance (WPOI, tumor budding, tumor cell nest size, tumor-stroma ratio, stromal lymphocyte infiltration and stroma type) were classified in Table 2 and shown in Figure 1. As the number of categories increases and the number of cases per category decreases, the power of the statistical tests will decrease, so the statistical analysis was performed by reducing the parameters to two subgroups.

When the relationship of six new histopathological parameters, whose prognostic importance was investigated, with demographic and routine parameters, the following results were obtained.

WPOI: There was a significant relationship between WPOI and lymph node metastasis. It was observed that WPOI 5 had more lymph node metastases than WPOI 4 (*p* = 0.01) (Table 3).

Tumor budding: Perineural invasion and extranodal extension were more common in cases with five or more tumor buds, which was statistically significant (*p* < 0.001 and *p* = 0.02, respectively). Cases with five or more tumor buds had more advanced pT and pN stages (*p* = 0.01 and *p* = 0.02, respectively) (Table 3). Perineural invasion (*p* = 0.049, HR: 6.832, %95 Cl:1.130–47.868) and extranodal extension (*p* = 0.022, HR: 9.443, %95 Cl:1.381–64.597) were identified as independent predictive factors by multivariate analysis.

Tumor cell nest size: Perineural invasion was more common and the pT stage was more advanced in cases whose tumor cell nest size was composed of a single cell (*p* < 0.001 and *p* = 0.02, respectively). The cases whose tumor cell size consisted of a single cell were in stage IV cases (*p* = 0.03) (Table 3). Perineural invasion (*p* = 0.001, HR: 10.278, %95 Cl:2.634–40.106) was identified as an independent predictive factor by multivariate analysis.

The follow-up period ranged from 1 to 155 months. The mean follow-up time was 44.6 months, and the median follow-up was 30 months. Of 73 patients, 28 (38.4%) died, and 45 (61.6%) were still alive. The two-year overall survival rate of all OSCC patients was 67.7%. Univariate overall survival analyses of all parameters were indicated in Table 5. The presence of perineural invasion and also lymph node metastasis were associated with poor prognosis. It was also statistically significant that the prognosis was worse as the pT, pN and stage were increased. The relationship between new parameters and the prognosis was made between the first formed groups and later created two-category subgroups of them. Although the cases showed a worse prognosis as the degree of WPOI increased, there was no statistical significance between WPOI and prognosis. It was seen that tumor budding was the most related parameter with prognosis. It was observed that the prognosis of the cases worsened as the number of tumor buds increased, and this was statistically significant (*p* < 0.001 and *p* = 0.02) (Table 5). The cases in which the smallest invasive cell nest consisted of a single cell in the stroma had a poor prognosis, and this was statistically significant (*p* = 0.02) (Table 5). Although there is lower survival in tumors with low stromal lymphocyte infiltration, stroma-rich pattern and immature types of stroma, no statistically significant results were found (Table 5).

Multivariate Cox regression survival analyses were performed for perineural invasion, presence of lymph node metastasis, stage, tumor budding and tumor cell nest size. The perineural invasion was the independent prognostic factor for survival (*p* < 0.001, HR:2.976, %95 Cl:1.317–6.729).

## 4. Discussion

The most common site of head and neck squamous cell carcinomas is the oral cavity [24]. Local recurrence has been reported between 10 and 40% in early stage cancers [25,26]. Recognition of prognostic factors associated with low survival rate and local recurrence and their indication in pathology reports are important for treatment management [27]. The relationship between histological grade and prognosis is controversial. There are studies indicating that there is [28,29] or is not [30,31,32] a significant relationship between histological grade and prognosis. The reason for this is that differentiation is based mainly on keratinization. However, in addition to keratinization, nuclear pleomorphism and mitotic activity should also be considered during the evaluation. This ambiguous situation has driven research into parameters associated with the tumor microenvironment. In this study, the power of parameters related to tumor microenvironment in predicting prognosis was investigated in a cohort of 73 OSCC Cases.

Evaluation of the invasion pattern constitutes an important element of different scoring systems that have been proposed instead of histological grading [6,13,30,33]. The WPOI, which was updated and gained importance with the article published by Brandwein-Gensler et al., in 2005, is one of them [6]. WPOI was added to the ICCR (International Collaboration on Cancer Reporting) carcinomas of the oral cavity histopathology reporting guide published in 2018, and it is recommended as an optional report component in the CAP guideline [34]. In the study published by Brandwein-Gensler et al., WPOI 4 (HR.2.0 95%CI 1.62–8.77, *p* = 0.004) and WPOI 5 (HR:6.4, 95%CI 2.43–13.97, *p* = 0.001) had lower overall survival. Furthermore, WPOI 5 cases have been shown to have a higher risk of local recurrence compared to WPOI 4 (*p* = 0.015) [6]. Studies evaluating WPOI in oral cavity tumors have increased in recent years, and the literature is more consistent than the WHO histological grading. WPOI 4 and 5 were associated with many poor prognostic factors such as bone invasion, nodal metastases, locoregional recurrence, and low disease-free survival [30,31,33,35,36]. In our study, although there was no statistically significant difference in overall survival, WPOI 5 was associated with more frequent lymph node metastasis and advanced stage at the time of diagnosis.

Tumor budding is one of the prognostic factors widely acknowledged in colon cancers and is recommended to be reported in the CAP colon cancer guideline. Studies have shown that tumor budding is a promising histopathological parameter for many cancers [27,37,38,39,40]. Tumor budding is thought to reflect tumor discohesion, motility and invasion ability and promote oncogenesis. Tumor budding is a dynamic process that begins with the separation of the tumor cell from the main tumor mass. Studies show that E-cadherin, a regulator protein of epithelial-mesenchymal transition, plays an important role in this process. Loss of E-cadherin is associated with a decrease in β-catenin in the cell membrane and/or cytoplasm [41]. Studies on tumor budding in oral cavity carcinomas are relatively recent compared to colorectal carcinoma. Therefore, tumor budding is not included in oral cavity tumor reporting protocols/datasets. In the meta-analysis of Almangush et al., which included 16 studies evaluating the prognostic significance of tumor budding in oral cavity tumors, tumor budding was associated with overall survival (HR: 1.88, 1.25–2.82%95%CI), lymph node metastasis (OR: 7.08, 1.75–28.73 95%CI) and disease-free survival (HR: 1.83, 1.34–2.50 95%CI) [42]. The studies in this meta-analysis classified tumor budding into two tiers, and the limit value varied between 3 and 10. In our study, although a four-tiered classification was made at first, the limit value of five was accepted later. In the meta-analysis, the rate of tumors containing five or more buds ranged from 26.1% to 51.7%, while it was 36.9% in our study. Tumor budding is an important predictor of overall survival in either four-tier or two-tier classification schemes. As per our study, high tumor budding is associated with poor OS in OSCC [35,43,44,45].

Tumor cell nest size is related to poor prognosis in cancers like lung, oesophagal and urothelial carcinomas [11,12,37]. A study by Kadota et al. which contains 485 lung SCC patients showed that single-cell invasion was a poor prognostic factor for overall survival in multivariate analyzes [37]. Later, Weichert et al. proposed a staging system including tumor budding and cell nest size [46]. Boxberg et al. applied this staging system to 157 OSCC patients and found that it was associated with overall survival similar to lung SCC [14].

Perineural invasion and stage were associated with tumor cell nest size in our study and only perineural invasion was an independent predictive factor for tumor cell nest size. A single-cell invasion pattern was found to be a statistically significant prognostic factor in our study.

The association between tumor stroma ratio and prognosis has also been reported in several cancers [47,48,49]. Tumor-stroma ratio may also be a morphological change induced by epithelial-mesenchymal transition [18,22,50]. A low tumor-stroma ratio (stroma-rich) was significantly associated with poor survival in many studies [22,43,51].

Immune cells are one of the components of the tumor microenvironment, because of this the immune infiltrations associated with tumors have been studied for a time [19]. The immune response of the tumor is related to the biological response of the tumor. Tumors with the same tumor differentiation, stage or another histological parameter may have completely different immune responses. Therefore, the percentage of the stromal lymphocytic infiltration of the tumor is an important parameter in cancer prognosis [52]. Heikkenen et al. showed that perineural invasion is associated with low lymphocytic infiltration but not with grade and stage [19]. It has been proven that the tumor microenvironment is important in the invasion and metastasis of many cancers, and it has been found to seriously affect the prognosis. It is known that cancer-associated fibroblasts form the extracellular matrix containing large amounts of collagen and the interaction between stromal cells and tumor cells has biological effects on OSCC progression and metastasis [53]. It is known that the stromal component in the invasive area of the tumor is keloid-like or myxoid, indicating the biological behavior of the tumor in many cancers, such as the colon, esophagus, etc. [11,15]. It has been reported that patients with immature stroma have a more aggressive clinical course.

In Luo’s article, which approaches the cancer microenvironment from different perspectives, nasopharyngeal carcinoma is defined as an “ecological and evolutionary unity” disease. He stated that the ecological interaction between cancer cells and their microenvironment is a dynamic process, and they are in a mutual relationship. Cancer consists of cells that show heterogeneity within themselves, and the relationships of these different cells with each other and with the microenvironment are defined as ecological interactions and are governed by many cytokines and extracellular vesicles. Therefore, cancer has not only a genetic but also an ecological and evolutionary development [54].

One of the limitations of our study is that our cohort size was limited, and the follow-up period of some of our cases was short. Since it is more important to predict the prognosis of early stage tumors, only early stage tumors were included in some of the studies [45]. We concluded that tumor budding and single-cell invasion should be considered prognostic histopathologic parameters in OSCC pathology reports. These two parameters may be included in adjuvant chemotherapy criteria. By studying these histopathological parameters in larger cohorts with longer follow-up times and conducting meta-analyses, a better agreement can be made about the histopathological parameters that should be included in the pathology reports.

## 5. Conclusions

Tumor budding reflects the aggressiveness of the tumor and represents invasion and metastasis and poor OS. It is easily identified by pathologists in HE sections and is a good predictor of recurrence, metastasis and survival. Since it is supported by many articles in this field, tumor budding is a promising tissue-based biomarker for prognosis and should be reported as a microscopic biomarker in routine practice. Having a successful histopathological parameter to predict the prognosis of patients will have a significant contribution to planning the treatment of patients in tumor councils.

## Figures and Tables

**Table 1:** The distribution of demographic and histopathological parameters of the cases.

Variable		Number of Patients (%)
Age	<65	35 (47.9%)
	≥65	38 (52.1%)
Gender	Female	36 (49.3%)
	Male	37 (50.7%)
Tumor localization	Tongue	42 (57.5%)
	Buccal mucosa	26 (35.6%)
	Floor of mouth	4 (5.5%)
	Palate	1 (1.4%)
Tumor size	0–2 cm	38 (52.1%)
	>2 cm	35 (47.9%)
Histological grade	Well-differentiated	11 (15.1%)
	Moderately differentiated	55 (75.3%)
	Poorly differentiated	7 (9.6%)
Depth of invasion	0–5 mm	32 (43.8%)
	>5 mm	41 (56.2%)a
Surgical margin	Negative	41 (56.2%)
	Positive	32 (43.8%)
Perineural invasion	Absent	55 (75.3%)
	Present	18 (24.7%)
Lymphovascular invasion	Absent	71 (97.3%)
	Present	2 (2.7%)
Lymph node dissection	Absent	18 (24.7%)
	Present	55 (75.3%)
Lymph node metastasis	Absent	27 (49.1%)
	Present	28 (50.9%)
Extranodal extension	Absent	15 (53.6%)
	Present	13 (46.4%)
Size of extranodal extension	0–2 mm	8 (61.5%)
	>2 mm	5 (38.5%)
pT	T1	22 (30.1%)
	T2	34 (46.6%)
	T3	11 (15.1%)
	T4	6 (8.2%)
pN	N0	45 (61.6%)
	N1	5 (6.8%)
	N2	21 (28.8%)
	N3	2 (2.7%)
AJCC stage	Stage I	18 (24.7%)
	Stage II	24 (32.9%)
	Stage III	6 (8.2%)
	Stage IV	25 (34.2%)
Locoregional recurrence	Absent	54 (74%)
	Present	19 (26%)
Distant metastasis	Absent	67 (91.8%)
	Present	6 (8.2%)

**Table 2:** The distribution of histopathological parameters was examined for prognostic significance.

Variable		Number of Patients (%)
Worst pattern of invasion (WPOI)	WPOI 2	1 (1.4%)
	WPOI 3	9 (12.3%)
	WPOI 4	50 (68.5%)
	WPOI 5	13 (17.8%)
Tumor budding	Absent	20 (27.4%)
	1–4 buds	26 (35.6%)
	5–9 buds	12 (16.4%)
	≥10 buds	15 (20.5%)
Tumor cell nest size	>15 cells	10 (13.7%)
	5–15 cells	10 (13.7%)
	2–4 cells	20 (27.4%)
	Single-cell	33 (45.2%)
Tumor-stroma ratio	Stroma-poor	26 (35.6%)
	Stroma-rich	47 (64.4%)
Stromal lymphocyte infiltration	Low (0–30%)	24 (32.9%)
	Intermediate (31–60%)	30 (41.1%)
	High (61–100%)	19 (26%)
Stroma type	Mature	55 (75.3%)
	Immature	18 (24.7%)

**Table 3:** The relationship between the histopathological parameters was examined for prognostic significance (worst pattern of invasion, tumor budding, tumor cell nest size) and the routine histopathological parameters and demographic parameters.

Variable	Worst Pattern of İnvasion			Tumor Budding			Tumor Cell Nest Size		
	4	5	*p*	0–4 Buds	≥5 Buds	*p*	>1 Cell	Single Cell	*p*
Age									
<65	24 (48%)	8 (61.5%)	0.53	20 (43.5%)	15 (55.6%)	0.34	18 (45%)	17 (51.5%)	0.64
≥65	26 (52%)	5 (38.5%)		26 (56.5%)	12 (44.4%)		22 (55%)	16 (48.5%)	
Gender									
Female	23 (46%)	8 (61.5%)	0.36	20 (43.5%)	16 (59.3%)	0.23	18 (45%)	18 (54.5%)	0.48
Male	27 (54%)	5 (38.5%)		26 (56.5%)	11 (40.7%)		22 (55%)	15 (45.5%)	
Tumor size									
0–2 cm	26 (52%)	7 (53.8%)	1	25 (54.3%)	13 (48.1%)	0.63	21 (52.5%)	17 (51.5%)	1
>2 cm	24 (48%)	6 (46.2%)		21 (45.7%)	14 (51.9%)		19 (47.5%)	16 (48.5%)	
Depth of invasion									
0–5 mm	20 (40%)	3 (23.1%)	0.34	23 (50%)	9 (33.3%)		22 (55%)	10 (30.3%)	0.06
>5 mm	30 (60%)	10 (76.9%)		23 (50%)	18 (66.7%)		18 (45%)	23 (69.7%)	
Histological grade									
Well	6 (12%)	0	0.35	8 (17.4%)	3 (11.1%)	0.12	8 (20%)	3 (9.1%)	0.18
Moderately	38 (76%)	12 (92.3%)		36 (78.3%)	19 (70.4%)		30 (75%)	25 (75.8%)	
Poorly	6 (12%)	1 (7.7%)		2 (4.3%)	5 (18.5%)		2 (5%)	5 (15.2%)	
Surgical margin									
Negative	30 (60%)	5 (38.5%)	0.21	27 (58.7%)	14 (51.9%)	0.63	24 (60%)	17 (51.5%)	0.48
Positive	20 (40%)	8 (61.5%)		19 (41.3%)	13 (48.1%)		16 (40%)	16 (48.5%)	
Perineural invasion									
Absent	38 (76%)	7 (53.8%)	0.16	42 (91.3%)	13 (48.1%)	<0.001	37 (92.5%)	18 (54.5%)	<0.001
Present	12 (24%)	6 (46.2%)		4 (8.7%)	14 (51.9%)		3 (7.5%)	15 (45.5%)	
Lymphovascular invasion									
Absent	49 (98%)	12 (92.3%)	0.37	45 (97.8%)	26 (96.3%)	1	39 (97.5%)	32 (97%)	1
Present	1 (2%)	1 (7.7%)		1 (2.2%)	1 (3.7%)		1 (2.5%)	1 (3%)	
Lymph node metastasis									
Absent	22 (53.7%)	1 (10%)	0.01	16 (53.3%)	11 (44%)	0.59	15 (57.7%)	12 (41.4%)	0.28
Present	19 (46.3%)	9 (90%)		14 (46.7%)	14 (56%)		11 (42.3%)	17 (58.6%)	
Extranodal extension									
Absent	10 (52.6%)	5 (55.6%)	1	11 (78.6%)	4 (28.6%)	0.02	8 (72.7%)	7 (41.2%)	0.21
Present	9 (47.4%)	4 (44.4%)		3 (21.4%)	10 (71.4%)		3 (27.3%)	10 (58.8%)	
Size of extranodal extension									
0–2 mm	4 (44.4%)	1 (25%)	1	2 (66.7%)	3 (30%)	0.51	2 (66.7%)	3 (30%)	0.51
>2 mm	5 (55.6%)	3 (75%)		1 (33.3%)	7 (70%)		1 (33.3%)	7 (70%)	
pT									
T1	14 (28%)	3 (23.1%)	0.21	16 (34.8%)	6 (22.2%)	0.01	14 (35%)	8 (24.2%)	0.02
T2	25 (50%)	4 (30.7%)		23 (50%)	11 (40.7%)		21 (52.5%)	13 (39.4%)	
T3	8 (16%)	3 (23.1%)		7 (15.2%)	4 (14.8%)		5 (12.5%)	6 (18.2%)	
T4	3 (6%)	3 (23.1%)		0	6 (22.2%)		0	6 (18.2%)	
pN									
N0	31 (62%)	4 (30.8%)	0.12	32 (69.6%)	13 (48.1%)	0.02	29 (72.5%)	16 (48.5%)	0.08
N1	4 (8%)	1 (7.7%)		3 (6.5%)	2 (7.4%)		3 (7.5%)	2 (6.1%)	
N2	14 (28%)	7 (53.8%)		11 (23.9%)	10 (37%)		8 (20%)	13 (39.4%)	
N3	1 (2%)	1 (7.7%)		0	2 (7.4%)		0	2 (6.1%)	
AJCC stage									
Stage I	10 (20%)	3 (23.1%)	0.15	13 (28.3%)	5 (18.5%)	0.12	12 (30%)	6 (18.2%)	0.03
Stage II	18 (36%)	1 (7.7%)		17 (37%)	7 (25.9%)		15 (37.5%)	9 (27.3%)	
Stage III	5 (10%)	1 (7.7%)		5 (10.9%)	1 (3.7%)		5 (12.5%)	1 (3%)	
Stage IV	17 (34%)	8 (61.5%)		11 (23.9%)	14 (51.9%)		8 (20%)	17 (51.5%)	
AJCC stage									
Stage I–II	28 (56%)	4 (30.8%)	0.12	30 (65.2%)	12 (44.4%)	0.09	27 (67.5%)	15 (45.5%)	0.09
Stage III–IV	22 (44%)	9 (69.2%)		16 (34.8%)	15 (55.6%)		13 (32.5%)	18 (54.5%)	
Locoregional recurrence									
Absent	37 (74%)	10 (76.9%)	1	33 (71.7%)	21 (77.78%)	0.78	29 (72.5%)	25 (75.8%)	0.79
Present	13 (26%)	3 (23.1%)		13 (28.3%)	6 (22.2%)		11 (27.5%)	8 (24.2%)	
Distant metastasis									
Absent	46 (92%)	11 (84.6%)	0.59	44 (95.7%)	23 (85.2%)	0.18	38 (95%)	29 (87.9%)	0.4
Present	4 (8%)	2 (15.4%)		2 (4.3%)	4 (14.8%)		2 (5%)	4 (12.1%)	

Tumor-stroma ratio: The tumors with a depth of invasion greater than 5 mm were rich in the stroma and this was statistically significant (*p* = 0.02) (Table 4); Stromal lymphocytic infiltration: Tumors with stromal lymphocyte infiltration rate between 0 and 30% had more perineural invasion (*p* = 0.03) (Table 4); Stroma type: Tumor size >2 cm, tumors with poorly differentiated morphology and extranodal extension were more likely to be seen as immature stroma, and this was statistically significant (*p* = 0.02, *p* = 0.01 and *p* = 0.01, respectively) (Table 4). An extranodal extension was identified as an independent predictive factor by multivariate analysis.

**Table 4:** The relationship between the histopathological parameters examined for prognostic significance (tumor-stroma ratio, stromal lymphocyte infiltration, stroma type) and the routine histopathological parameters and demographic parameters.

Variable	Tumor-Stroma Ratio			Stromal Lymphocyte İnfiltration			Stroma Type		
	Stroma-Poor	Stroma-Rich	*p*	0–30%	31–100%	*p*	Mature	Immature	*p*
Age									
<65	10 (38.5%)	25 (53.2%)	0.32	15 (62.5%)	20 (40.8%)	0.13	23 (41.8%)	12 (66.7%)	0.1
≥65	16 (61.5%)	22 (46.8%)		9 (37.5%)	29 (59.2%)		32 (58.2%)	6 (33.3%)	
Gender									
Female	13 (50%)	23 (48.9%)	1	13 (54.2%)	23 (46.9%)	0.62	29 (52.7%)	7 (38.9%)	0.41
Male	13 (50%)	24 (51.1%)		11 (45.8%)	26 (53.1%)		26 (47.3%)	11 (61.1%)	
Tumor size									
0–2 cm	14 (53.8%)	24 (51.1%)	1	10 (41.7%)	28 (57.1%)	0.31	33 (60%)	5 (27.8%)	0.02
>2 cm	12 (46.2%)	23 (48.9%)		14 (58.3%)	21 (42.9%)		22 (40%)	13 (72.2%)	
Depth of invasion									
0–5 mm	16 (61.5%)	16 (34%)	0.02	7 (29.2%)	25 (51%)	0.08	27 (49.1%)	5 (27.8%)	0.19
>5 mm	10 (38.5%)	31 (66%)		17 (70.8%)	24 (49%)		28 (50.9%)	13 (72.2%)	
Histological grade									
Well	7 (26.9%)	4 (8.5%)	0.09	3 (12.5%)	8 (16.3%)	0.09	10 (18.2%)	1 (5.6%)	0.01
Moderately	16 (61.5%)	39 (83%)		16 (66.7%)	39 (79.6%)		43 (78.2%)	12 (66.7%)	
Poorly	3 (11.5%)	4 (8.5%)		5 (20.8%)	2 (4.1%)		2 (3.6%)	5 (27.8%)	
Surgical margin									
Negative	14 (53.8%)	27 (57.4%)	0.8	10 (41.7%)	31 (63.3%)	0.08	30 (54.5%)	11 (61.1%)	0.78
Positive	12 (46.2%)	20 (42.6%)		14 (58.3%)	18 (36.7%)		25 (45.5%)	7 (38.9%)	
Perineural invasion									
Absent	22 (84.6%)	33 (70.2%)	0.27	14 (58.3%)	41 (83.7%)	0.03	44 (80%)	11 (61.1%)	0.12
Present	4 (15.4%)	14 (29.8%)		10 (41.7%)	8 (16.3%)		11 (20%)	7 (38.9%)	
Lymphovascular invasion									
Absent	26 (100%)	45 (95.7%)	0.53	22 (91.7%)	49 (100%)	0.1	54 (98.2%)	17 (94.4%)	0.43
Present	0	2 (4.3%)		2 (8.3%)	0		1 (1.8%)	1 (5.6%)	
Lymph node metastasis									
Absent	9 (52.9%)	18 (47.4%)	0.77	10 (50%)	17 (48.6%)	1	21 (53.8%)	6 (37.5%)	0.37
Present	8 (47.1%)	20 (52.6%)		10 (50%)	18 (51.4%)		18 (46.2%)	10 (62.5%)	
Extranodal extension									
Absent	4 (50%)	11 (55%)	1	3 (52.6%)	12 (55.6%)	0.11	13 (72.2%)	2 (20%)	0.01
Present	4 (50%)	9 (45%)		7 (47.4%)	6 (44.4%)		5 (27.8%)	8 (80%)	
Size of extranodal extension									
0–2 mm	2 (50%)	3 (33.3%)	0.56	3 (42.9%)	2 (33.3%)	0.72	2 (40%)	3 (37.5%)	0.51
>2 mm	2 (50%)	6 (66.7%)		4 (57.1%)	4 (66.7%)		3 (60%)	5 (62.5%)	
pT									
T1	9 (34.6%)	13 (27.7%)	0.62	5 (20.8%)	17 (34.7%)	0.05	19 (34.5%)	3 (16.7%)	0.07
T2	13 (38.2%)	21 (44.7%)		11 (45.8%)	23 (46.9%)		26 (47.3%)	8 (44.4%)	
T3	2 (7.7%)	9 (19.1%)		3 (12.5%)	8 (16.3%)		8 (14.5%)	3 (16.7%)	
T4	2 (7.7%)	4 (8.5%)		5 (20.8%)	1 (2%)		2 (3.6%)	4 (22.2%)	
pN									
N0	18 (69.2%)	27 (57.4%)	0.7	14 (58.3%)	31 (63.3%)	0.25	37 (67.3%)	8 (44.4%)	0.05
N1	1 (3.8%)	4 (8.5%)		1 (4.2%)	4 (8.2%)		4 (7.3%)	1 (5.6%)	
N2	7 (26.9%)	14 (29.8%)		7 (29.2%)	14 (28.6%)		14 (25.5%)	7 (38.9%)	
N3	0	2 (4.3%)		2 (8.3%)	0		0	2 (11.1%)	
AJCC stage									
Stage I	7 (26.9%)	11 (23.4%)	0.69	5 (20.8%)	13 (26.5%)	0.21	16 (29.1%)	2 (11.1%)	0.16
Stage II	10 (38.5%)	14 (29.8%)		8 (33.3%)	16 (32.7%)		19 (34.5%)	5 (27.8%)	
Stage III	1 (3.8%)	5 (10.6%)		0	6 (12.2%)		5 (9.1%)	1 (5.6%)	
Stage IV	8 (30.8%)	17 (36.2%)		11(45.8%)	14 (28.69%)		15 (27.3%)	10 (55.6%)	
AJCC stage									
Stage I–II	17 (65.4%)	25 (53.2%)	0.44	13 (54.2%)	29 (59.2%)	0.87	35 (63.6%)	7 (38.9%)	0.11
Stage III–IV	9 (34.6%)	22 (46.8%)		11 (45.8%)	20 (40.8%)		20 (36.4%)	11 (61.1%)	
Locoregional recurrence									
Absent	18 (69.2%)	36 (76.6%)	0.58	20 (83.3%)	34 (69.4%)	0.32	39 (70.9%)	15 (83.3%)	0.36
Present	8 (30.8%)	11 (23.4%)		4 (16.7%)	15 (30.6%)		16 (29.1%)	3 (16.7%)	
Distant metastasis									
Absent	25 (96.2%)	42 (89.4%)	0.41	21 (87.5%)	46 (93.9%)	0.38	51 (92.7%)	16 (88.9%)	0.63
Present	1 (3.8%)	5 (10.6%)		3 (12.5%)	3 (6.1%)		4 (7.3%)	2 (11.1%)	

**Table 5:** Univariate overall survival analyses of all parameters.

		2 Year OS	HR (95%CI)	*p*
Age	<65	77.50%	1	0.09
	≥65	58.60%	1.890 (0.873–4.093)	
Gender	Female	76.70%	1	0.32
	Male	58.50%	1.456 (0.686–3.091)	
Tumor localization	Tongue	68.90%	1	0.99
	Other localization	65.60%	0.996 (0.47–2.109)	
Tumor size	0–2 cm	80.50%	1	0.11
	>2 cm	55.10%	1.823 (0.848–3.918)	
Histological grade	Well-differentiated	54.50%	1	0.17
	Moderately differentiated	74.30%	0.752 (0.279–2.028)	
	Poorly differentiated	42.90%	1.865 (0.539–6.456)	
Depth of invasion	0–5 mm	73.10%	1	0.4
	>5 mm	63.60%	1.388 (0.639–3.013)	
Surgical margin	Negative	72.90%	1	0.21
	Positive	58.70%	1.348 (0.769–2.361)	
Perineural invasion	Absent	75.60%	1	<0.001
	Present	43%	3.272 (1.517–7.056)	
Lymphovascular invasion	Absent	68.20%	1	0.15
	Present	50%	2.697(0.635–11.452)	
Lymph node metastasis	Absent	73.70%	1	0.03
	Present	56.90%	2.368 (1.029–5.448)	
Extranodal extension	Absent	60.20%	1	0.94
	Present	53.80%	1.037(0.361–2.977)	
Size of extranodal extension	0–2 mm	60%	1	0.99
	>2 mm	50%	0.993(0.215–4.599)	
pT	T1	75.70%	1	0.01
	T2	75.5	1.272(0.483–3.349)	
	T3	45	1.920(0.573–6.429)	
	T4	33	6.044 (1.608–22.710)	
pN	N0	74.7	1	0.04
	N1	60	1.238(0.275–5.579)	
	N2	57	2.900 (1.315–6.400)	
	N3	50	3.246 (0.410–25.709)	
Stage	Stage I–II	75.30%	1	0.03
	Stage III–IV	57.80%	2.220 (1.046–4.710)	
Locoregional recurrence	Absent	72.70%	1	0.04
	Present	54.20%	2.166 (0.999–4.699)	
Distant metastasis	Absent	71.40%	1	0.01
	Present	22.20%	3.226 (1.197–8.691)	
WPOI	WPOI 2–3	80%	1	0.17
	WPOI 4	67.10%	2.358 (0.548–10.144)	
	WPOI 5	58.20%	4.106 (0.821–20.533)	
WPOI	WPOI 4	67.10%	1	0.26
	WPOI 5	58.20%	1.682 (0.659–4.292)	
Tumor budding	Absent	82.60%	1	<0.001
	1–4 buds	78.20%	1.534 (0.513–4.580)	
	5–9 buds	61.90%	1.471 (0.394–5.499)	
	≥10 buds	35.90%	4.786 (1.622–14.119)	
Tumor budding	0–4 buds	79.90%	1	0.02
	≥5 buds	47%	2.252 (1.068–4.748)	
Tumor cell nest size	>15 cells	80%	1	0.15
	5–15 cells	87.50%	1.685 (0.280–10.153)	
	2–4 cells	84.40%	1.904 (0.395–9.184)	
	Single-cell	47.60%	3.627 (0.829–15.870)	
Tumor cell nest size	>1 cell	83.30%	1	0.02
	Single-cell	47.60%	2.252 (1.060–4.781)	
Tumor-stroma ratio	Stroma-poor	73.80%	1	0.33
	Stroma-rich	64.40%	1.324 (0.561–3.124)	
Stromal lymphocyte infiltration	0–30%	52.10%	1	0.25
	31–60%	70.40%	0.593 (0.255–1.378)	
	61–100%	82.60%	0.482 (0.180–1.289)	
Stromal lymphocyte infiltration	0–30%	52.10%	1	0.1
	31–100%	75%	0.546 (0.258–1.156)	
Stroma type	Mature	69.50%	1	0.38
	Immature	62.50%	0.581 (0.252–1.336)	

## Data Availability

You can access the data by contacting the authors of the study.
